# Risk Factors for *Legionella longbeachae* Legionnaires’ Disease, New Zealand 

**DOI:** 10.3201/eid2307.161429

**Published:** 2017-07

**Authors:** Emma Kenagy, Patricia C. Priest, Claire M. Cameron, Debbie Smith, Pippa Scott, Vicki Cho, Peter Mitchell, David R. Murdoch

**Affiliations:** University of Otago, Christchurch, New Zealand (E. Kenagy, P. Scott, V. Cho, D.R. Murdoch);; Canterbury District Health Board, Christchurch (E. Kenagy, D. Smith, P. Mitchell);; University of Otago, Dunedin, New Zealand (P. Priest, C.M. Cameron)

**Keywords:** Legionnaires’ disease, *Legionella longbeachae*, risk factors, compost, gardening, parasites, New Zealand, case–control study, COPD, chronic obstructive pulmonary disease, smoking, respiratory infections, pneumonia

## Abstract

*Legionella longbeachae*, found in soil and compost-derived products, is a globally underdiagnosed cause of Legionnaires’ disease. We conducted a case–control study of *L. longbeachae* Legionnaires’ disease in Canterbury, New Zealand. Case-patients were persons hospitalized with *L. longbeachae* pneumonia, and controls were persons randomly sampled from the electoral roll for the area served by the participating hospital. Among 31 cases and 172 controls, risk factors for Legionnaires’ disease were chronic obstructive pulmonary disease, history of smoking >10 years, and exposure to compost or potting mix. Gardening behaviors associated with *L. longbeachae* disease included having unwashed hands near the face after exposure to or tipping and troweling compost or potting mix. Mask or glove use was not protective among persons exposed to compost-derived products. Precautions against inhaling compost and attention to hand hygiene might effectively prevent *L. longbeachae* disease. Long-term smokers and those with chronic obstructive pulmonary disease should be particularly careful.

## Abstract 2

Gardeners with chronic obstructive pulmonary disease or a history of smoking should take extra precautions to prevent infection.

Legionnaires’ disease is a community-acquired severe pneumonia that causes substantial illness and death (*1*). Of the patients that are hospitalized with the disease, 30% require intensive care unit admission and ≈10% die (*1*,*2*). In most parts of the world, the majority of reported cases are caused by *Legionella pneumophila* (*1*), which sometimes contaminates water (e.g., water from cooling towers) and causes outbreaks. However, Legionnaires’ disease is often underdiagnosed because the tests required are not usually performed unless specifically requested. In addition, a substantial diagnostic bias exists in reported cases globally because the most commonly used test, the urinary antigen test, detects only *L. pneumophila* serogroup 1 (*2*). 

In 2010, in Christchurch, New Zealand, implementation of an enhanced testing strategy that included PCR testing of respiratory specimens led to a >4-fold increase in the number of detected cases of Legionnaires’ disease (*2*). New Zealand has the highest reported incidence of Legionnaires’ disease in the world (*1*), and in part because of the more rigorous testing in this country, the epidemiology of detected Legionnaires’ disease in New Zealand differs from that of most other countries; a high proportion of cases are caused by *L. longbeachae* (*3*–*5*). *L. longbeachae* can be found in soil and compost-derived products (*6*–*9*). In 2013, a total of 51% of reported Legionnaires’ disease cases were caused by *L. longbeachae*, 28% by *L. pneumophila*, and 21% by other *Legionella* species (*3*).

Two small case–control studies on risk factors for *L. longbeachae* pneumonia conducted in South Australia have been published (*10*,*11*). These studies found that preexisting cardiac or respiratory disease, long-term smoking, gardening, exposure to hanging baskets, using potting mix, and eating or drinking after gardening without washing hands were risk factors for this disease. O’Connor et al. suggested that both inhalation and ingestion were potential modes of transmission and advised that long-term smokers and those with respiratory and cardiac conditions should take particular care of their hygiene during and after gardening (*11*).

We conducted a population-based, case–control study to assess the importance of various risk factors for *L. longbeachae* pneumonia hospitalization in a setting with high case ascertainment to build on the South Australia studies and inform public health efforts to prevent Legionnaires’ disease. Our main aim was to identify gardening behaviors or types of exposure to potting mix and compost that are associated with the increased risk for Legionnaires’ disease. In addition, we assessed several other possible risk factors that could facilitate the introduction of organisms from compost into the lungs.

## Methods

### Setting

This study was conducted in the region of Canterbury, New Zealand, which has a population of ≈530,000 (*12*). Canterbury contains the city of Christchurch, also known as The Garden City, as well as horticultural and agricultural areas. The Canterbury District Health Board is the publicly funded healthcare provider for the region and includes the local public health unit (Community and Public Health) to which all cases of Legionnaires’ disease must be notified. The study was conducted over the course of 2 summers during the peak of Legionnaires’ disease activity (October 1, 2013–March 31, 2014 and October 1, 2014–March 31, 2015). Because no cases of Legionnaires’ disease had been previously reported in this district in persons <30 years of age, the study population was persons >30 years of age who were listed on the electoral roll for the electorates with boundaries within the region served by the Canterbury District Health Board. This project was approved by the University of Otago Human Ethics Committee (H13/065).

### Cases

Cases of pneumonia in which the patient was hospitalized and disease onset occurred during peak Legionnaires’ season were considered for inclusion into this study. Only confirmed cases that were positive by culture or by PCR for *L. longbeachae* or had a >4-fold increase in reciprocal *L. longbeachae* antibody titers were eligible for inclusion (*2*).

### Controls

To obtain controls, before each summer, a random sample that was frequency-matched to the expected age distribution of case-patients was taken from the electoral roll. The list of potential controls was randomly sorted and broken into 3 groups; each group was sent an invitation to participate in the study at approximately monthly intervals starting in mid-September in 2013 and at the end of October in 2014. This strategy was used in an effort to interview controls at a rate similar to that of the case-patients. We aimed to recruit 3 controls per case-patient. If no response was received to the first letter, a follow-up letter was sent. If no response was received and the person appeared in the telephone book at the address on the electoral roll, we followed up by phone call. 

### Data Collection

All notified cases of Legionnaires’ disease are followed up by a Health Protection Officer (HPO) from the Public Health Unit. Follow-up usually includes a face-to-face interview. During the enrollment period, case-patients were invited to take part in our study at these interviews. If they were willing to participate and were on the electoral roll, they were given the usual questionnaire augmented by a structured questionnaire designed specifically for the study that included more detailed questions about possible relevant exposures. For the period of the study, Legionnaires’ disease cases were followed up by 1 of the authors (D.S.) or an HPO who had been trained (for consistency purposes) to administer the study questionnaire.

Controls who agreed to take part were telephoned by 1 of the authors (E.K., V.C., or P.S.) or a trained research assistant (an HPO). Those who reported diarrhea, fever, chest pain, or cough lasting >24 hours in the previous 3 weeks were excluded.

The study questionnaire contained questions on demographics, smoking status, and preexisting health conditions. Questions were also asked about garden environment; garden activities; pets; and exposure to soil, compost, and potting mix.

### Analysis

We analyzed data using Stata 13 (*13*). All calculations of odds ratios (ORs) used a logistic mixed model that adjusted for age (a continuous variable) and included period (October 1, 2013–March 31, 2014 or October 1, 2014–March 31, 2015) as a random effect. We interpreted ORs as measures of relative risk.

For exposures to purchased compost and potting mix, the products were combined (and referred to as purchased compost products or simply as compost) because their manufacturing processes are similar and because we grouped them together to help maximize precision. We assessed the effects of behaviors that have been encouraged as protective (*14*) (e.g., wearing a mask, wearing gloves, and wetting compost before use) in compost users only. We stratified estimation of the OR of exposure to compost by smoking status to assess whether smoking modifies the effect of exposure to compost.

We identified 2 groups of behaviors that could potentially explain the increased risk for Legionnaires’ disease through using compost: behaviors that increase the risk for aerosolizing compost organisms (moving compost around, opening compost bags, and using compost indoors) and behaviors that increase the risk of getting compost organisms near or in the mouth (eating or drinking, smoking, or touching the face after using compost without washing hands). We assumed that both of these types of activities could result in inhalation of *Legionella*, and we conducted a multivariable analysis to assess the relative importance of causal paths that included aerosolization and hand-to-face transfer in explaining the increased risk associated with the use of compost.

We used a causal diagram (*15*) as a guide for multivariable analyses (Technical Appendix Figure). Aerosolization of compost and hand-to-face transfer of compost are intermediate factors between the use of compost, which is known to be a risk factor for Legionnaires’ disease, and diagnosis of Legionnaires’ disease. Our study had insufficient data to precisely estimate these intermediate effects or to definitively determine whether a direct effect of compost remained after adjusting for both aerosolization and hand-to-face activities (which would suggest that other causal pathways in addition to aerosolization or hand-to-face activities exist). However, our assumption was that if the OR associated with compost use reduced to close to 1 in a multiple regression model that included aerosolization and hand-to-face variables, this finding would indicate that we had identified the main causal pathways (*16*).

We did not have enough data to put all the separate relevant variables into a single multivariable model. Our initial intention was to create 1 composite variable indicating experience of any of the aerosolizing activities and 1 variable indicating any of the hand-to-face activities and to include those composite variables in a multivariable model along with compost use. However, all of the case-patients who had used compost in the 3 weeks previous to getting sick had also engaged in >1 of the aerosolizing activities, so that composite variable could not be included in the multivariable model. Therefore, we selected 1 of the aerosolization variables as a representative aerosolizing activity as follows. First, we constructed separate models that each included compost use and one of the aerosolizing activities. We then chose the model, and therefore the activity, with the lowest Akaike information criterion (a measure of the relative quality of statistical models) (*17*). This activity was then included in a model with compost use and the composite hand-to-face variable to determine whether there was any direct effect of compost use after adjustment for these 2 mechanisms of exposure. Population attributable fractions, which can be interpreted as the percentage by which disease incidence would be reduced if the risk factor was eliminated, were calculated for these variables from this model (*18*).

## Results

Thirty-seven cases of *L. longbeachae* Legionnaires’ disease were initially notified to Community and Public Health during the study period (24 in the first summer and 13 in the second). One case was excluded because the patient was not on a Canterbury electoral roll, and 2 patients were interviewed but later excluded from the analysis when *L. longbeachae* infection was not confirmed. Three case-patients declined to participate in the study, yielding an overall response rate of 31 out of 34 (91%) for eligible cases.

Each summer, letters were sent to 301 potential controls (total 602 potential controls) identified from the electoral roll. In total, 214 potential controls (36% of all potential controls and 46% of those who could be contacted) agreed to take part in the study (Figure). Of these, 10 were ineligible, 172 were interviewed, 6 were unable to be interviewed, and 26 were not interviewed for administrative reasons. In the first study period, interviewing stopped after 69 controls had been interviewed (when 3 controls/case was reached), but afterward 2 case-patients who did not have *L. longbeachae* disease were excluded. For the second study period, all controls who agreed to take part were interviewed, except for the 6 whose consent was confirmed too late for the interview to take place.

**Figure:**
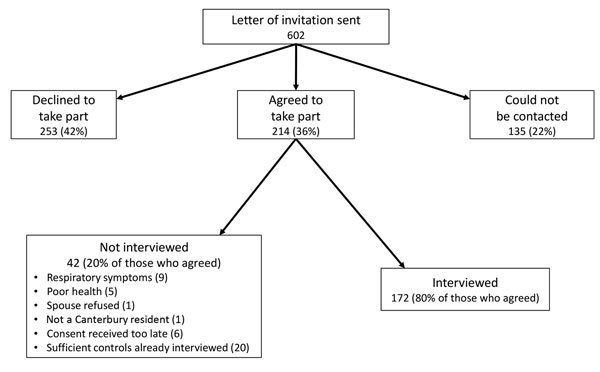
Flowchart of solicitation and participation of controls for study of *Legionella longbeachae* Legionnaires’ disease, New Zealand, October 1–March 31, 2013–2014 and 2014–2015.

### Demographic Characteristics, Health Conditions, and Smoking

Case-patients were slightly older and more likely than controls to be men (Table 1). The health condition most strongly associated with Legionnaires’ disease was chronic obstructive pulmonary disease (COPD; OR 4.2, 95% CI 1.2–14.7) (Table 2). Smoking was associated with an increased risk for Legionnaires’ disease, and a dose-dependent response was evident.

**Table 1:** Demographic characteristics of Legionnaires’ disease case-patients and controls, New Zealand, October 1–March 31, 2013–2014 and 2014–2015

Characteristic	Case-patients, no. (%)	Controls, no. (%)
Age, y		
30–39	1 (3)	3 (2)
40–49	4 (13)	8 (5)
50–59	4 (13)	36 (21)
60–69	7 (23)	56 (33)
70–79	10 (32)	49 (28)
>80	5 (16)	20 (12)
Median age	69	66
Sex		
M	18 (58)	74 (43)
F	13 (42)	98 (57)
Ethnicity*		
European	30 (97)	156 (91)
Māori	1 (3)	6 (3)
Pacific	0	1 (1)
Other	0	8 (5)
Not specified	0	1 (1)
Income		
≤$70,000†	19 (61)	83 (48)
>$70,000	6 (19)	53 (31)
Missing	6 (19)	36 (21)
Possible occupational exposure‡		
Yes	2 (6)	7 (4)
No	28 (90)	165 (96)
Missing	1 (3)	

*If Māori and other ethnicities were ticked, the person’s ethnicity was recorded as Māori; if Pacific but not Māori was ticked, the person’s ethnicity was recorded as Pacific. †$70,000 was approximately the reported New Zealand median household income in 2013 (*19*). ‡For case-patients: 1 garden center worker and 1 farm manager. For controls: 4 farmers, 1 soil scientist, 1 gardener, and 1 landscaper.

**Table 2:** Univariate analyses of the associations between health conditions or smoking and *Legionella longbeachae* Legionnaires’ disease, New Zealand, October 1–March 31, 2013–2014 and 2014–2015*

Health conditions	Case-patients, no. (%)	Controls, no. (%)	OR (95% CI)
Preexisting health conditions			
Cardiac disease	8 (26)	35 (20)	1.2 (0.47–3.0)
Respiratory disease	10 (32)	35 (20)	2.1 (0.87–4.9)
Asthma	8 (26)	28 (16)	1.9 (0.76–4.9)
COPD	5 (16)	9 (5)	4.2 (1.2–14.7)
Diabetes	5 (16)	12 (7)	2.8 (0.88–9.0)
Immunosuppression	6 (19)	16 (9)	2.7 (0.91–7.9)
Smoking			
Current smoker	4 (13)	9 (5)	2.4 (0.66–8.5)
Ever smoked	19 (61)	63 (37)	2.6 (1.2–5.7)
Smoking period†			
Never smoked	12 (39)	109 (65)	1.0
Smoked <20 y	5 (16)	26 (16)	1.6 (0.5–5.1)
Smoked 20 y to <40 y	4 (13)	17 (10)	2.3 (0.6–8.0)
Smoked >40 y	10 (32)	15 (9)	5.6 (2.0–16.0)

*COPD, chronic obstructive pulmonary disease; OR, odds ratio. †Length of time smoking could not be determined for 5 control respondents. Percentages are given for those in which length of time smoking was known.

### Gardening Environment and Pets

All case-patients and all but 4 controls had an outdoor garden on their property, but case-patients were 3 times more likely than controls to have an indoor garden (defined as a glass or tunnel house, hydroponic system, or conservatory) (Table 3). Almost all case-patients had gardened during the 3 weeks before becoming ill, and almost all controls had gardened during the 3 weeks before being interviewed. Using purchased compost products was strongly associated with Legionnaires’ disease (OR 6.2, 95% CI 2.2–17.3) and using homemade compost was not. Case-patients were also 3 times as likely as controls to own >1 cat.

**Table 3:** Univariate analyses of the associations between garden type, garden exposures, or pets and *Legionella longbeachae* Legionnaires’ disease, New Zealand, October 1–March 31, 2013–2014 and 2014–2015*

Environmental factors	Case-patients, no. (%)	Controls, no. (%)	OR (95% CI)
Garden type			
Outdoor garden on property	31 (100)	168 (98)	
Enclosed garden on property†	11 (36)	29 (17)	3.0 (1.3–7.1)
Hanging pots or baskets on property	10 (32)	53 (31)	0.95 (0.41–2.2)
Garden exposures			
Near dripping, hanging pots or baskets	4 (13)	37 (22)	0.55 (0.18–1.7)
Gardened in the past 3 weeks	29 (94)	151 (88)	1.9 (0.42–8.7)
Spent any time gardening outdoors	28 (90)	150 (87)	1.3 (0.36–4.7)
Spent any time gardening indoors‡	10 (32)	54 (31)	1.0 (0.45–2.4)
Used purchased compost	26 (84)	84 (49)	6.2 (2.2–17.3)
Used homemade compost§	7 (23)	40 (22)	1.0 (0.40–2.6)
Pets			
Own dog(s)	7 (23)	40 (23)	0.97 (0.36–2.6)
Own cat(s)	19 (61)	63 (37)	3.0 (1.3–6.8)
Own bird(s)	4 (13)	12 (7)	1.9 (0.55–6.5)

*OR, odds ratio. †Glasshouse, tunnel house, hydroponics, or conservatory. ‡Including tending to potted plants. §Irrespective of use of purchased compost.

Behaviors such as moving the hands to the face before handwashing (OR 4.8) and aerosolizing compost (OR 9.9) were associated with the risk for disease (Table 4). None of the behaviors that have been considered possibly protective against Legionnaires’ disease were associated with a reduction in disease risk.

**Table 4:** Univariate analyses of the associations between gardening behaviors and *Legionella longbeachae* Legionnaires’ disease, New Zealand, October 1–March 31, 2013–2014 and 2014–2015*

Activities performed around the time of gardening	Case-patients, no. (%)	Controls, no. (%)	OR (95% CI)
Hand to face after using compost and before washing hands			
Ate or drank	7 (23)	13 (8)	4.1 (1.4–11.8)
Touched face	12 (39)	31 (18)	3.6 (1.5–8.6)
Smoked	2 (7)	5 (3)	2.2 (0.39–12.0)
Any opportunity for getting hands near face (smoking, eating or drinking, or touching face) before washing hands	16 (52)	36 (21)	4.8 (2.1–11.1)
Possible compost aerosolization			
Opened compost	21 (68)	55 (32)	5.2 (2.2–12.1)
Used compost indoors	7 (23)	9 (5)	6.6 (2.1–20.7)
Tipped or troweled compost	24 (77)	60 (35)	8.3 (3.2–21.5)
Moved compost with hands	15 (48)	52 (30)	2.6 (1.1–5.8)
Purchased compost or moved potting mix around (with hands or by tipping/troweling)	26 (84)	70 (41)	9.9 (3.4–28.3)
Possible protective factors†			
Wore a mask while using compost	5 (19)	12 (14)	1.5 (0.46–4.8)
Wore gloves while handling compost	17 (65)	50 (60)	1.2 (0.49–3.1)
Wet compost down before use	4 (13)	18 (11)	1.6 (0.48–5.4)

*OR, odds ratio. †Among only those who had used compost in previous 3 weeks.

Among participants who had smoked for >10 years, those who had used compost had an OR for Legionnaires’ disease of 7.9 (95% CI 1.5–43.2); among those who had not smoked or smoked for <10 years but used compost, the OR was 4.6 (95% CI 1.2–17.2). Although these CIs overlap, these results suggest an increased effect of compost use on the risk for Legionnaires’ disease in persons who have smoked long-term. The OR for compost users who had smoked for >10 years compared with those with neither risk factor was 14.7 (95% CI 3.7–58.4).

In multivariable regression, including compost use and each of the aerosolization variables separately, the model with tipping and troweling compost had the lowest Akaike information criterion (158), and so tipping and troweling was included in a multivariable model with the hand-to-face variable (Table 5). With these 2 variables in the model, the OR for compost use (the direct effect) was 0.97, and the ORs for the indirect effects of compost use through tipping and troweling (6.1) and hand-to-face contact (2.3) were lower than their univariate ORs (8.3 and 4.8, respectively; Table 4) but still raised (). CIs were wide, as expected given the small sample size. The population attributable fractions calculated from this multivariable model were 65% for tipping and troweling compost and 30% for making hand-to-face contact before handwashing.

**Table 5:** Multivariable analyses of the direct and intermediate effects of types of compost use on *Legionella longbeachae* Legionnaires’ disease, New Zealand, October 1–March 31, 2013–2014 and 2014–2015*

Compose use risk factor	OR (95% CI)
Use of compost in previous 3 weeks	0.97 (0.16–5.9)
Tip or trowel compost	6.1 (1.3–29.4)
Hand to face before handwashing	2.3 (0.88–6.1)

*OR, odds ratio.

## Discussion

Forty years since the first identification of Legionnaires’ disease, we still do not have a thorough understanding of how to prevent disease caused by the species *L. longbeachae*. Risk factors for Legionnaires’ disease include host characteristics and modifiable behavioral or environmental factors. Most published data have focused on the risk factors for *L. pneumophila* Legionnaires’ disease: smoking, older age, chronic cardiovascular or respiratory disease, immunosuppression, and exposure to water aerosols (*1*). Data on the risk factors for *L. longbeachae* disease are lacking, beyond the presumed association with compost exposure. Our study highlights some similarities and differences with risk factors for Legionnaires’ disease caused by *L. pneumophila*. We found that COPD and smoking are host risk factors for *L. longbeachae* disease. Those who had ever smoked were twice as likely to get *L. longbeachae* disease as those who had never smoked, and the risk was higher for those who had smoked longer. Having COPD, although uncommon (16% of case-patients and 5% of controls), increased disease risk 4-fold.

Use of purchased compost products during the reference period was the strongest exposure risk factor for *L. longbeachae* disease in univariate analysis, and those who had smoked for >10 years were more likely than nonsmokers to get Legionnaires’ disease after using purchased compost products. The mechanisms by which compost use increases the risk for disease appear to include the activities that increase the likelihood of its aerosolization and activities that could lead to compost being transferred to the face or mouth, presumably leading to inhalation of particles contaminated with *Legionella* bacteria. Our findings suggest that the biggest reduction in *L. longbeachae* disease could be made by eliminating activities that aerosolize compost (population attributable fraction 65%). We also found that hand hygiene might be a useful measure for preventing *L. longbeachae* disease, with the potential to reduce disease by 35%. However, we did not demonstrate a benefit from wearing gloves or masks while gardening.

Our findings are similar to those from the previous studies conducted in South Australia (*10*,*11*) in identifying that having ever smoked, having a history of long-term smoking, recently using compost, and eating or drinking after using compost products before washing hands are risk factors for *L. longbeachae* disease. However, our study did not confirm that preexisting cardiac illness or being near dripping hanging pots or baskets were associated with *L. longbeachae* disease.

We found a previously unidentified association between cat ownership and *L. longbeachae* infection. The association was unaffected by including smoking or use of compost products in the multivariable model (data not shown). However, given the relatively small sample size and potential for a false-positive finding, further investigation of this association is required before any definitive conclusions can be made.

The small sample size was a limitation of this study. Having low numbers of cases resulted in wide CIs for ORs and the limited ability to perform more detailed multivariable analyses. Assessing the effect of different compost handling methods and different compost product exposures on disease risk was not possible. Another limitation was the poor response rate among controls. It is possible that respondents differed from the general population (e.g., a selection for gardeners probably occurred). Because information about the nature of the study provided to participants noted that *L. longbeachae* is found in soil, nongardeners might have perceived that the study investigators were only interested in interviewing gardeners and declined to participate. The lack of nongardeners in the study could cause an underestimation of the effect of gardening-related behaviors on disease risk. On the other hand, recall bias, in which case-patients overestimate their exposure to risks, could cause overestimation of the effect. The precision of this study did not warrant formal quantitative bias analysis (*20*).

Although we demonstrated no protective effect with use of gloves or masks while gardening, we are reluctant to advise against these practices on the basis of this study alone, especially given the increased disease risk associated with activities that lead to the aerosolization of compost and activities that can lead to the transfer of compost from the hand to the face. Those using face masks and gloves while handling purchased compost products should use single-use disposable masks and wash hands thoroughly after removing gloves but before removing and disposing of the mask; not washing hands before mask removal might be a potential exposure to the face. Smokers (both current smokers and those with a long-term history of smoking) should be made aware that they are at increased risk for Legionnaires’ disease and advised to be particularly careful when using purchased compost products.

Transmission of *L. longbeachae* bacteria to humans is still not fully understood, and it is not possible to use compost without moving it around. However, public health messages should encourage persons handling purchased compost products and potting mix to minimize aerosolization (for example, opening bags away from the face and keeping the compost close to the ground when moving it) and to keep hands away from the face until they have been thoroughly washed. Larger studies to assess the risk associated with particular compost-associated behaviors more fully could better inform prevention strategies.

Technical Appendix Causal diagram showing the relationship between compost use and Legionnaires’ disease.
