# Supplementary material for: Risk Factors for Legionella longbeachae Legionnaires’ Disease, New Zealand
Source: Emerg Infect Dis. 2017 Jul;23(7):1148–54. doi: 10.3201/eid2307.161429 (PMC5512494; doi:10.3201/eid2307.161429)
Supplement: Technical Appendix — Causal diagram showing the relationship between compost use and Legionnaires’ disease. [file 16-1429-Techapp-s1.pdf]

Article DOI: https://dx.doi.org/10.3201/eid2307.161429

Risk Factors for *Legionella longbeachae* Legionnaires' Disease, New Zealand

Technical Appendix

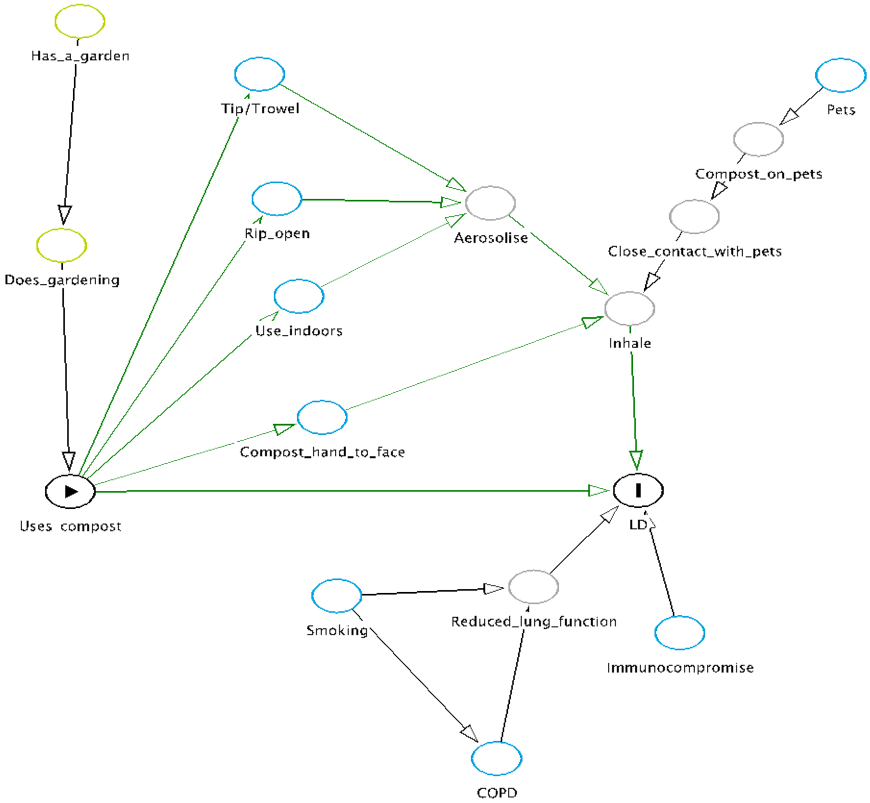

Technical Appendix Figure. Causal diagram for relationship between compost use and Legionnaires' disease. Causal diagrams (directed acyclic graphs) represent the relationships between different variables that might affect the exposure and outcome of interest (*1*). This diagram, made by using the Web-based program DAGitty (*2*), makes clear that smoking and COPD are not confounders of the relationship between compost use and Legionnaires' disease; no relationship between smoking and COPD and compost use was found. The gray nodes are unobserved parts of the causal pathways between different variables (blue) and the outcome (LD). The diagram also makes clear that we assume for our analyses that gardening has an effect on the risk for Legionnaires' disease solely through the use of compost. COPD, chronic obstructive pulmonary disease; LD, Legionnaires' disease.

References

1. Shrier I, Platt RW. Reducing bias through directed acyclic graphs. BMC Med Res Methodol. 2008;8:70. PubMed http://dx.doi.org/10.1186/1471-2288-8-70

2. Textor J, Hardt J, Knüppel S. DAGitty: a graphical tool for analyzing causal diagrams. Epidemiology. 2011;22:745. PubMed http://dx.doi.org/10.1097/EDE.0b013e318225c2be
